# Subpacket structure in strong VLF chorus rising tones: characteristics and consequences for relativistic electron acceleration

**DOI:** 10.1186/s40623-021-01467-4

**Published:** 2021-07-07

**Authors:** John C. Foster, Philip J. Erickson, Yoshiharu Omura

**Affiliations:** 1 MIT Haystack Observatory, Westford, MA 01886 USA; 2 Research Institute for Sustainable Humanosphere, Kyoto University, Kyoto, Japan

**Keywords:** VLF chorus, Subpackets, Radiation belt, Nonlinear interaction, Electron acceleration

## Abstract

Van Allen Probes in situ observations are used to examine detailed subpacket structure observed in strong VLF (very low frequency) rising-tone chorus elements observed at the time of a rapid MeV electron energization in the inner magnetosphere. Analysis of the frequency gap between lower and upper chorus-band waves identifies *f*_ceEQ_, the electron gyrofrequency in the equatorial wave generation region. Initial subpackets in these strong chorus rising-tone elements begin at a frequency near 1/4 *f*_ceEQ_ and exhibit smooth gradual frequency increase across their > 10 ms temporal duration. A second much stronger subpacket is seen at frequencies around the local value of 1/4 *f*_ce_ with small wave normal angle (< 10°) and steeply rising d*f*/d*t*. Smooth frequency and phase variation across and between the initial subpackets support continuous phase trapping of resonant electrons and increased potential for MeV electron acceleration. The total energy gain for individual seed electrons with energies between 100 keV and 3 MeV ranges between 2 and 15%, in their nonlinear interaction with a single chorus element.

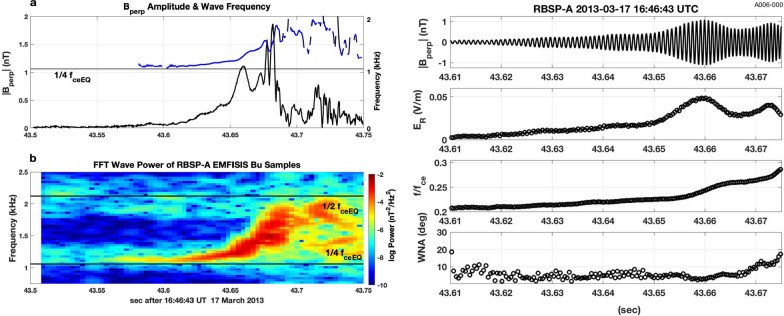

## Introduction

Rapid recovery (30 min; 10 × increase) of Earth’s radiation belt MeV electron fluxes following a storm-induced dropout have been reported (Baker et al. 2014; Foster et al 2014). Such rapid acceleration is associated with low-energy (10s–100s keV) electron injection, local acceleration (Reeves et al. 2013), and strong VLF chorus rising tones (risers) (Thorne et al. 2013). Jaynes et al. (2015) have shown that two distinct electron populations resulting from magnetospheric substorm activity are essential for the overall mechanism leading to acceleration of highly relativistic electrons in the outer belt: (1) a source population of injected tens of keV electrons that drive VLF wave growth, and (2) a seed population at hundreds of keV electrons that then are accelerated by these VLF waves to MeV energies. The structure and acceleration efficiency of the chorus wave elements is important for understanding this process. The Van Allen Probes twin spacecraft (Mauk et al. 2012) were well positioned in the inner magnetosphere to observe the characteristics of the chorus waves involved in the 17 March 2013 radiation belt recovery event (Baker et al. 2014; Foster et al. 2014).

## Subpacket structure in chorus wave elements

A typical chorus emission consists of a coherent wave with rising frequency (rising-tones). Each chorus element is composed of a sequence of discrete subpackets, each spanning a few to several 10 s of wave cycles. In general, each subpacket is characterized by smoothly increasing and decreasing wave amplitude, good coherence, and regularly varying wave frequency (e.g., Santolik et al. 2014). In the present manuscript, we discuss the coherence of the wave as observed by the resonant particles. For good coherence, a resonant particle is influenced only by a single wave. Chorus elements are divided at frequencies near half the electron gyrofrequency, 1/2 *f*_ce_, into lower and upper frequency bands (Tsurutani and Smith 1974). The initial part of the chorus emission at lower frequency results from linear instability driven by a temperature anisotropy which develops during electron injection into the inner magnetosphere. When the growing wave amplitude attains a threshold amplitude (Omura et al. 2009; Omura and Nunn 2011), the wave packet grows through a nonlinear process associated with the formation of an electromagnetic electron hole in velocity phase space (Omura et al. 2008). The source of the wave packet growth is primarily the frequency variation induced by the electron hole. For nonlinear chorus wave growth, the inhomogeneity factor, *S*, is important (*S* ~ − 0.4 for optimal wave amplitude). *S* depends linearly on d*f*/d*t*, d*B*_o_/d*x* and inversely on wave amplitude (Omura et. al., 2008). For a monotonic wave (d*f*/d*t* ~ 0), nonlinear wave growth due to formation of an electron hole does not take place near the equator where d*B*_o_/d*x* ~ 0.

At the equatorial source region, where d*B*_o_/d*x* is small, nonlinear wave growth is directly proportional to d*f*/d*t*. As the wave packet propagates away from the equator, its frequency structure remains the same, while wave amplitude increases with d*B*_o_/d*x* in a convective wave growth scenario. Once the wave amplitude exceeds the optimum wave amplitude (Omura and Nunn, 2011), the wave growth saturates, and the amplitude decreases gradually. In general, new seed waves are generated upstream from the equator by approaching resonant electrons with velocity towards the equator and whose phase has been modulated with the frequency of the preceding saturated wave packet. This packet has by definition a frequency higher than that of the original triggering wave. In the resulting interaction, the phase-modulated electrons generate a new triggering wave at the higher frequency. This wave growth process is repeated many times, forming a chorus element with a sequence of subpackets at progressively higher frequencies (Shoji and Omura, 2013). We note an important feature of this overall sequence: the wave frequency at any point in the chorus element is fixed in the near-equatorial generation region and does not vary as the wave packet propagates away from the equator. However, wave amplitude, as noted above, can vary as the wave propagates to its point of observation on an in situ platform. An extensive review of the nonlinear wave growth theory of whistler-mode chorus is presented by Omura (2021).

## Characteristics of strong subpackets

Santolik et al. (2014) found fine structure in chorus elements, with peak instantaneous amplitudes occasionally reaching up to 3 nT. That study also found the wave vector to be quasi-parallel to the background magnetic field for large-amplitude subpackets, with a distinct turn away from this direction when amplitudes are weaker. Zhang et al. (2019) found that subpackets with peak amplitudes > 1 nT have a < 1% occurrence rate. However, intense chorus subpackets are observed more frequently during highly disturbed (AE > 300 nT) events (Zhang et al. 2018). In a statistical analysis of intense lower‐band chorus wave subpackets, Zhang et al. (2020) investigated the relationships between wave frequency variations, packet length, and wave amplitude, and their temporal variability. They found that 15% of the wave power is carried by long subpackets with low-frequency sweep rates that agree well with the nonlinear theory of chorus wave growth. The remaining 85% of the wave power is associated with short packets with large frequency variations around the overall linear trend.

In this study, we use Van Allen Probes in situ observations to examine detailed subpacket structure observed in strong VLF rising-tone chorus elements seen at the time of a rapid (30 min) MeV electron energization in the inner magnetosphere at *L* ~ 4, previously discussed by Foster et al. (2014, 2017). We analyze local in situ wave electric and magnetic field observations made with the electric and magnetic field instrument and integrated science (EMFISIS) instrument (Kletzing^2012^), following the methods introduced by Foster et al. (2017). Based on our description of chorus wave subpacket formation, presented above, we identify a subpacket as a wave packet with smoothly increasing and decreasing wave amplitude and d*f*/d*t*, and with small (< 20°) or decreasing wave normal angle (WNA). The separation of individual subpackets is marked by a positive inflection in wave amplitude and d*f*/d*t* following an interval of decreasing wave amplitude and negative or reduced d*f*/d*t*. Other recent analyses of chorus wave structure (e.g., Zhang et al 2019; Zhang et al. 2020) have defined distinct subpackets as wave packets with a significant magnetic amplitude reduction between them. Because the spacecraft is situated at a non-zero magnetic latitude, the observations occur after the wave packets have propagated some distance from their origin in the equatorial region. As described below, both local and equatorial conditions are important for understanding the characteristics of observed subpackets, and therefore in the following analysis we differentiate between *f*_ce_, the local electron gyrofrequency measured at the point of observation, and *f*_ceEQ_, the equatorial electron gyrofrequency. Our determination of *f*_ceEQ_ is described below.

**Fig. 1:**
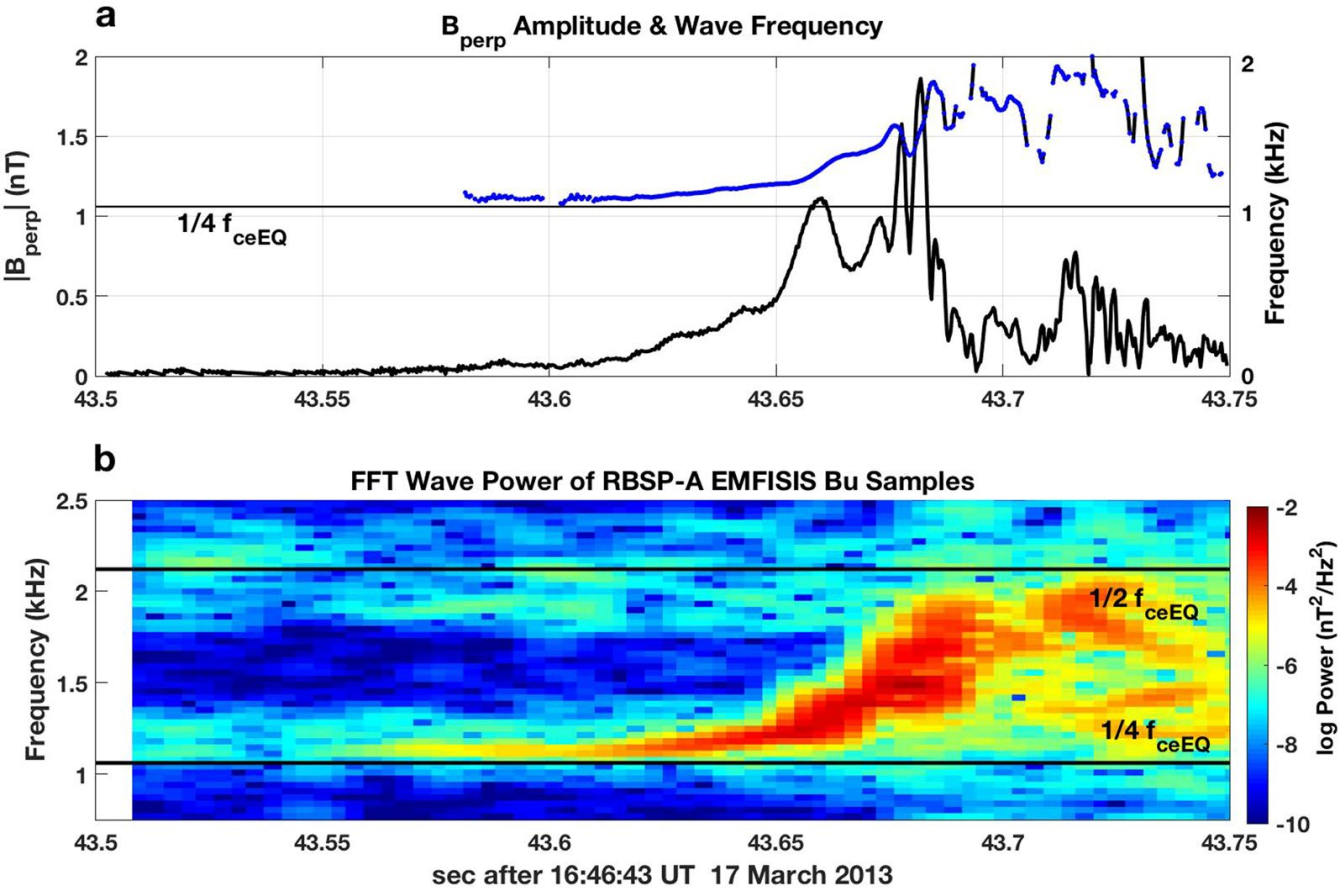
The analysis of Van Allen Probes EMFISIS high-frequency wave magnetic field observations of a strong VLF chorus element observed during the 17 March 2013 event reveals the typical subpacket structure and relationship between wave amplitude and frequency. The RBSP-A spacecraft was at *L* ~ 5, ~ 02 MLT, and at a position ~ 4° off the equatorial plane. **a** Wave frequency (blue) and perpendicular wave magnetic field amplitude (black) are shown across the multiple subpackets comprising the chorus element. **b** The wave magnetic field spectrogram shows an extended (> 80 ms) period of gradually increasing wave power and frequency before the onset of the first strong subpacket at 43.65 s

**Fig. 2:**
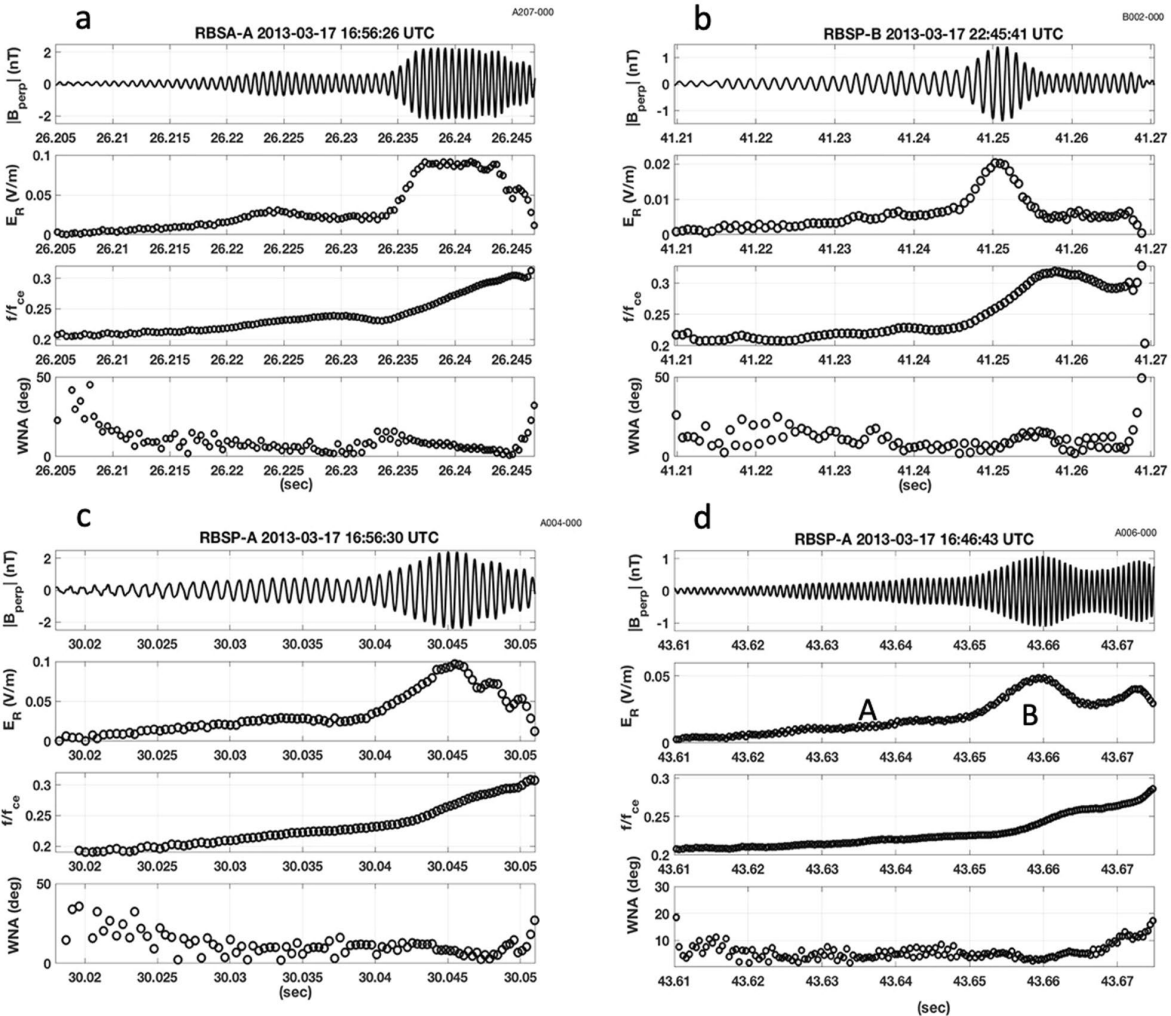
Detailed observations of the initial two subpackets of four strong chorus wave elements recorded during the March 17, 2013 event are shown in panels **a**–**d**. See text for description. Panel **b** displays the analysis for the chorus element shown in Fig. 1

Figure 1 presents Van Allen Probes EMFISIS observations of a strong VLF chorus element observed during the March 17, 2013 event previously discussed by Foster et al. (2014). The upper panel (a) shows the magnitude of the wave magnetic field perpendicular to the wave propagation direction, **k**, and the wave frequency determined over each 1/2 wave cycle. Panel (b) presents the wave power spectrogram for one component (Bu) of the chorus wave magnetic field. The strong chorus element in Fig. 1 began with an extended (> 50 ms) interval of weak wave amplitude with nearly constant frequency, preceding the onset of nonlinear wave growth. A sequence of subpackets with increasing wave frequency and variable amplitude followed. An initial, extended interval of weakly growing wave amplitude can be identified in ~ 50% of strong rising-tone chorus elements we have examined for strongly disturbed conditions during radiation belt electron recovery events (e.g., 14 Nov 2012, 1 March 2013 (14 UT), 17 March 2013 (16–17 UT), 17 March 2013 (22–23 UT). Observations during the March 17, 2013 event suggest that this initial period of wave growth occurred at or slightly below ~ ¼ *f*_ceEQ_ (see following discussion of how *f*_ce_EQ is determined). Figure 2 presents a wave property analysis of the initial subpackets for four strong chorus elements, typical of those observed while rapid MeV radiation belt electron acceleration was taking place during the event. For each of the individual chorus elements (a, b, c, d), the magnetic field waveform is shown in the upper panel, and shown sequentially below are the magnitude of E_R_, the normalized frequency (*f*/*f*_ce_), and the wave normal angle (WNA = acos (**k** • **B**_**0**_)). Wave frequency and wave normal angle are calculated at each 1/2 wave cycle following the procedure described by Foster et al. (2017). (Note that no frequency domain filtering has been applied to the observed waveform, with the result that stronger overlapping signals from other emissions could disrupt the wave cycle frequency analysis.)

In each case shown here, the onset of the first nonlinear subpacket is accompanied by a decrease of wave normal angle (WNA < 20°), is of extended duration (20–30 ms), exhibits slowly rising wave frequency and amplitude, and often begins near 1/4 *f*_*c*eEQ_. A second much stronger subpacket (see above) is seen at frequencies around 1/4 *f*_ce_ with low wave normal angle (WNA < 10°) and steeply rising d*f*/d*t*. For the cases shown in Fig. 2, wave frequency and phase vary smoothly both within the 1st and 2nd subpackets and across the transition between them. Peak wave magnetic field amplitudes of 1–2 nT (*E*_R_ ~ 50–100 mV/m) and subpacket durations of ~ 10 ms are typical for the strong 2nd subpackets. Additional descriptive characteristics of the subpackets denoted "A" and "B" in panel b are presented in Table 1.

**Table 1:** Subpacket characteristics

Parameter	A) 16:46:43 UT	B) 16:46:43 UT	C) 22:48:20 UT	D) 22:48:20 UT
*L*	5.13	5.13	6.49	6.49
*f*_ce_	5325 Hz	5325 Hz	3310 Hz	3310 Hz
ne	3 cm^−3^	3 cm^−3^	12 cm^−3^	12 cm^−3^
*f*_pe_	15,500 Hz	15,500 Hz	31,500 Hz	31,500 Hz
*f*_pe_/*f*_ce_	2.91	2.91	9.51	9.51
Magnetic latitude	− 4.17	− 4.17	− 2.54	− 2.54
# Cycles	46	21	55	19
Duration	39 ms	16 ms	73 ms	21 ms
*f*_ceEQ_	4200 Hz	4200 Hz	2900 Hz	2900 Hz
*f*/*f*_ce_	.206–.225	.225–.261	.208–.234	.218–.298
*f*/*f*_ceEQ_	.277–.303	.303–.351	.246–.277	.258–.352
|*E*_R_| max	20 mV/m	50 mV/m	6 mV/m	32 mV/m
*E*_res_ (*n* = 1)	60 keV	52 keV	10 keV	9 keV
500 keV % gain	2.45	5.22	2.30	1.38
1 MeV % gain	1.65	2.91	1.04	0.62

## Frequency span of lower band risers

**Fig. 3:**
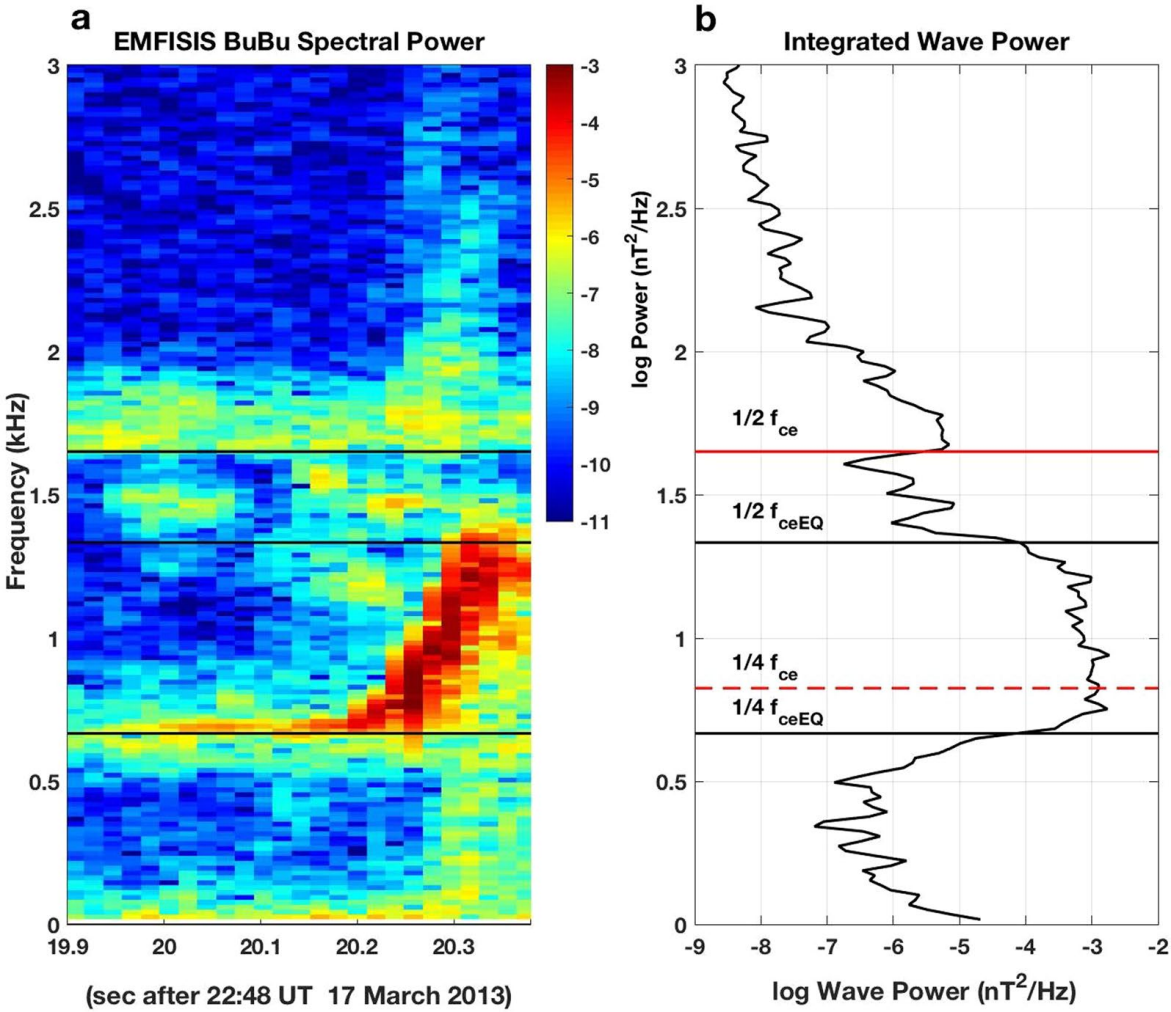
**a** The magnetic field spectrogram observed near L = 6.5 by Van Allen Probe B at 22:48:20 UT on 17 March 2013 exhibited clear damping at frequencies below 1/2 the local electron cyclotron frequency. **b** Integrated signal power at each frequency is shown for the ~ 400 ms of observations shown in **a**. The local value of *f*_ce_ is observed at the spacecraft and the equatorial value of *f*_ce_ is determined from the characteristics of the damping of the chorus element

At locations away from the equator, chorus elements are divided into lower and upper band emissions by a pronounced amplitude minimum below the local value 1/2 *f*_ce_ (Tsurutani and Smith 1974). This feature occurs as the wave packet propagates into regions of increasing magnetic field where damping at 1/2 *f*_ce_ progressively erodes wave amplitude at frequencies above 1/2 *f*_ceEQ_. The wave power damping seen in Fig. 3 between ½ the local and ½ the equatorial electron cyclotron frequency is in keeping with the nonlinear damping mechanism described by Hsieh and Omura (2018). Integrated signal power across the ~ 400 ms of observations shown in panel (a) clearly identifies 1/2 *f*_ceEQ_ in the equatorial chorus generation region as the point of onset of the ~ 1000 × decrease in the chorus element integrated wave power. Thus determined, the value of *f*_ceEQ_ also indicates that the frequency of initial chorus element wave growth occurred near 1/4 *f*_ceEQ_.

**Fig. 4:**
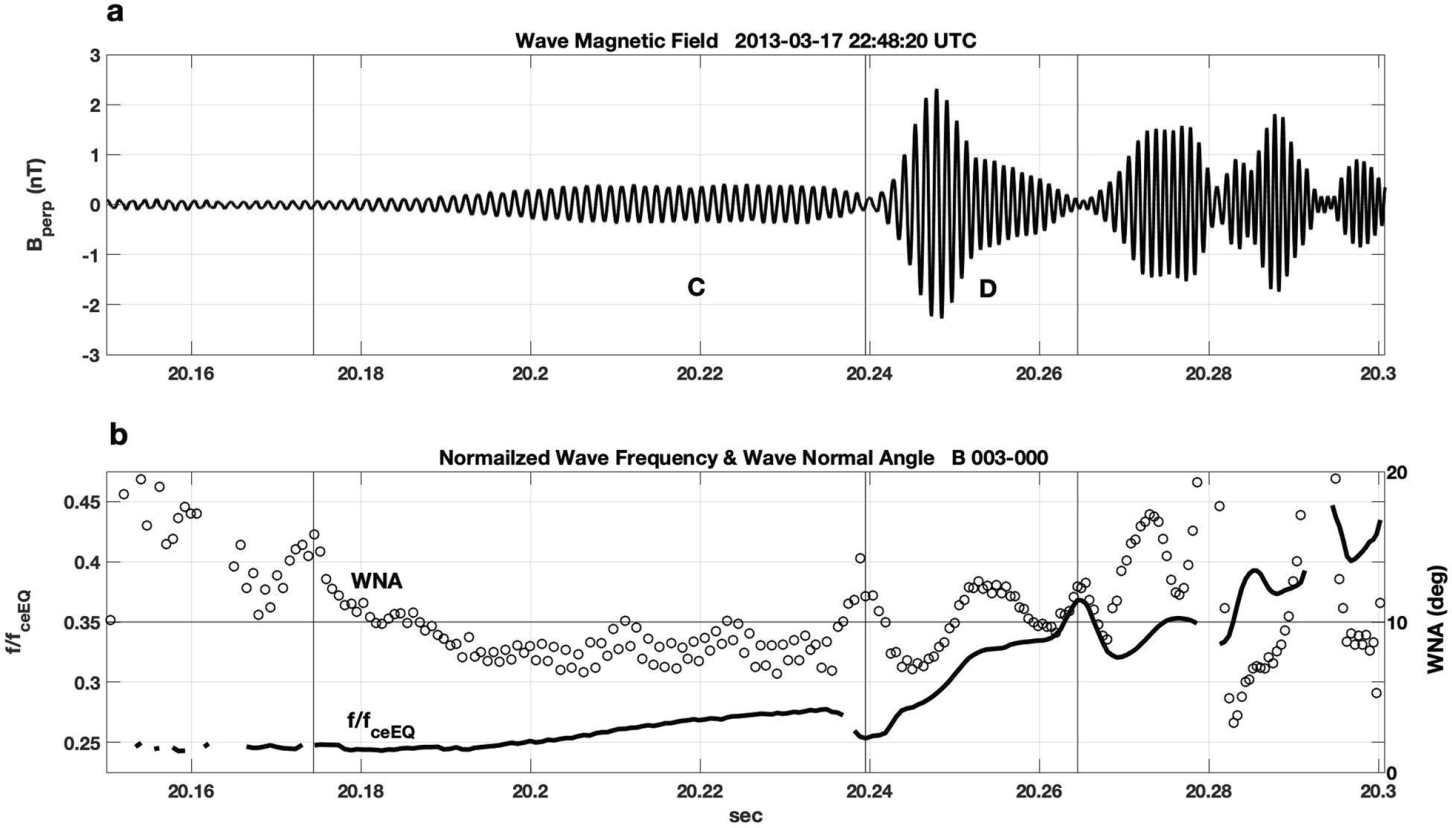
Analysis of the subpacket structure for the chorus element shown in Fig. 3 is shown. **a** Wave magnetic field exhibits an initial gradual increase in amplitude over < 70 ms, followed by a second strong subpacket with |B_perp_|~ 2 nT. **b** Normalized wave frequency (solid line, *f*/*f*_ceEQ_) and wave normal angle (open circles) are shown. Increasing wave amplitude and frequency are seen for WNA < 10°

Figure 4 presents a cycle-by-cycle analysis of the subpacket structure for the chorus element shown in Fig. 3. Many characteristics are similar to those discussed in Fig. 2. Here wave frequency normalized by *f*_ceEQ_ is shown. Coherent nonlinear wave growth and steadily increasing frequency began at 20.18 s near 1/4 *f*_ceEQ_ as WNA drops < 10°. The initial period of wave growth "C" extends for ~ 65 ms, followed by a 10-ms-strong 2nd subpacket "D". Wave amplitude decreased significantly between subpackets in this example, observed by RBSP-B during the same substorm injection event as the chorus element shown in Fig. 2b. The substorm events at 16 UT and 22 UT on March 17, 2013 were described by Foster et al (2017).

Table 1 enumerates and compares the characteristics and ambient conditions associated with chorus elements (A, B, C, and D) discussed above.

## Radiation belt effects

**Fig. 5:**
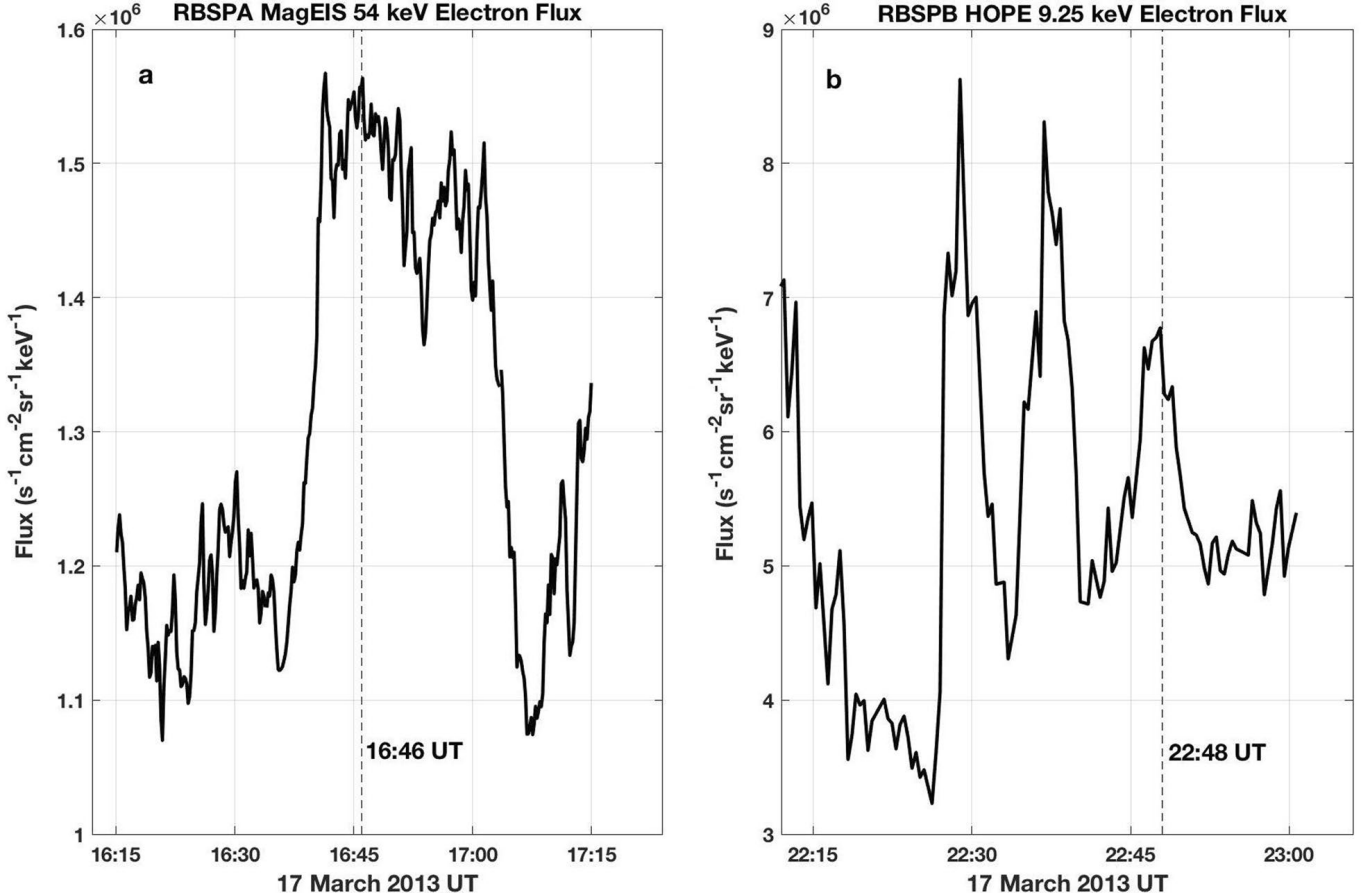
Sharp electron injections occurred at the (*n* = 1) cyclotron resonant energies associated with the growth of the chorus elements shown in Fig. 2 (**a** MagEIS 54-keV electrons) and Fig. 4 (**b** HOPE 9.25-keV electrons). The observation times of the chorus rising tones are indicated by dashed lines

The growth of rising-frequency chorus elements involves nonlinear cyclotron resonance (*n* = 1) with 10s to 100s keV electrons that are injected into the inner magnetosphere during substorm dipolarization events (e.g., Foster et al. 1976; Foster et al. 2014). As shown by analysis of simulations reproducing chorus emissions, the resonant current is mostly due to the nonlinear motion of resonant electrons moving slowly around the separatrix of the nonlinear trapping potential, resulting in formation of an electron hole. These untrapped resonant electrons are decelerated by the wave electric field, transferring energy to the wave. The nonlinear wave growth process by the electron hole has been demonstrated by self-consistent simulations (e.g., Omura et al. 2008; Hikishima et al. 2009) which conserve energy. During this interaction, perpendicular particle velocities and pitch angles are decreased substantially, such that these particles precipitate into the loss cone through their interaction with an individual chorus element (e.g., Foster 1973; Foster and Rosenberg 1976). Tsurutani et al. (2013) have noted that the rapid changes in pitch angle leading to electron microburst precipitation can only be explained through a coherent wave–particle interaction, rather than by quasilinear diffusion (e.g., Kennel and Petschek 1966). For the March 17, 2013 event described by Foster et al. (2014, 2017), strong chorus wave enhancement accompanied substorm events with onsets near 16 UT and 22 UT. Electron fluxes from the magnetic electron ion spectrometer (MagEIS; Blake et al.^ 2013^) and helium, oxygen, proton, and electron (HOPE) mass spectrometer (Funsten et al. 2013) instruments, shown in Fig. 5, characterized the discrete injections of electrons at the resonant energies (*n* = 1; 10–20° pitch angle) associated with the chorus elements described above in Figs. 2 and 4. For the event at ~ 16 UT, electron injection with energies ranging from 55 to 335 keV was observed. At the same time, the growing chorus waves serve as intermediaries for energy transfer from the lower energy injected particles to a pre-existing seed population of higher energy radiation belt electrons, as described by Jaynes et al. (2015). Prior to the electron injections and chorus wave growth, seed electrons with energies 200 keV–1 MeV were observed with fluxes significantly (> 10×) above detector threshold level, and were available for acceleration by the chorus waves.

The electrons giving energy to the wave are untrapped resonant electrons that form a hole in the velocity phase space. On the other hand, the electrons accelerated by the wave are trapped by the wave potential and their number density is much less than that of the untrapped resonant electrons. In the following, we discuss the acceleration efficiency of these trapped minority electrons which do not contribute much to the evolution of the wave packet. The wave growth process and the acceleration process are nearly independent.

Nonlinear interactions between 30 keV–several MeV seed electrons and chorus waves were investigated by Foster et al. (2017) using burst-mode EMFISIS observations of individual rising tone chorus elements as we have described above. That study concluded that nonlinear cyclotron resonance with parallel-propagating chorus waves assuming a 4% trapping probability could explain the rapid enhancement of MeV electron fluxes (10 × over 30 min) observed during the 17 March 2013 event. Such fast local acceleration of MeV electron fluxes as observed during that event is not solely responsible for the recovery of the outer radiation belt MeV electron population. The recovery time should be controlled by the occurrence rate and strength of the chorus emissions. If the injections of energetic electrons are not sufficient, the occurrence rate of strong chorus elements should decrease, and the recovery time would become longer (e.g., Hajra et al. 2015). Subsequent further acceleration accompanying inward diffusion over a period of days is well known to take place (e.g., Baker et al. 2014).

Hsieh and Omura (2018) and Omura et al. (2019) extended the theoretical analysis of the nonlinear energization potential of VLF chorus rising tones to the case of obliquely propagating waves (e.g., Santolik et al. 2009), including both cyclotron (*n* = 1) and Landau (*n* = 0) interactions. They found that nonlinear trapping of relativistic electrons by the Lorentz force of the perpendicular wave magnetic field resulted in effective electron acceleration (Omura et al. 2019). Furthermore, Higara and Omura (2020) investigated the nonlinear trapping of seed electrons with multiple subpackets within a single chorus element and described the finite probability of an electron being trapped (and accelerated) for 10s of milliseconds through multiple sequential subpackets.

**Fig. 6:**
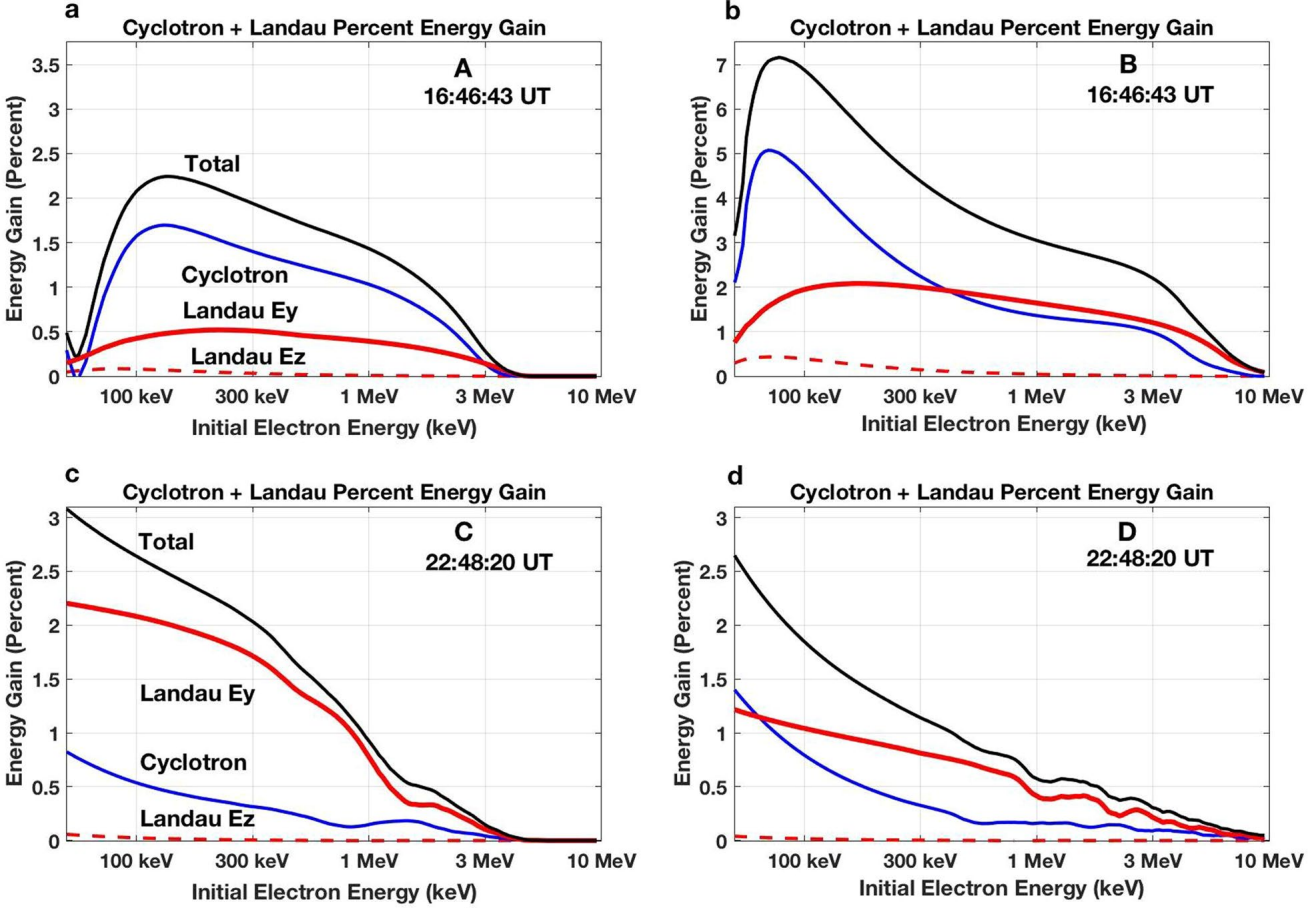
For obliquely propagating chorus waves, the nonlinear Lorentz force of the perpendicular wave magnetic field results in effective electron acceleration. The percent energy gained by seed electrons in a resonant interaction with a single chorus subpacket is shown following the calculations of Omura et al. (2019). **a**–**d** Give the separate results for the initial (A and C) and 2nd strong (B and D) subpackets of the chorus elements described in Fig. 2d (A and B) and 4 (C and D). Percent energy gain through the perpendicular Landau (*n* = 0) resonance is shown in red, through cyclotron (*n* = 1) resonance in blue, with the total gain shown in black

We apply the energy gain formulas developed by Omura et al. (2019) to individual riser subpackets (A, B, C, and D, denoted above in Figs. 2b and 4) during the 17 March 2013 event. These strong subpackets have temporal durations of 20–70 ms (20–50 wave cycles; see Table 1) and produce a maximum energy gain of 5–10 keV/wave cycle for trapped electrons with 1–3 MeV initial energy. Figure 6 presents the calculated percent energy gain for individual seed electrons with initial energies between 30 keV and 10 MeV during the separate first (gradual) or second (strong) nonlinear subpackets for each of the two chorus elements discussed above in Figs. 2b and 4.

Results indicate that for individual electrons resonant with the waves throughout a single subpacket (~ 10 ms), total energy increase of > 100 keV is possible. Energy increase from cyclotron (*n* = 1) and Landau (*n* = 0) effects is of similar magnitude, with Landau effects usually the larger contributor at relativistic energies. Individual 1-MeV seed electrons can experience a 1%–3% energy gain in their interaction with a single strong chorus element subpacket.

## Discussion

Prominent in our observations of strong chorus elements is a large-amplitude 2nd subpacket with steep d*f*/d*t* near 1/4 *f*_ce_, as measured at the spacecraft location. We suggest two causes for these characteristics. First, the wave group velocity maximizes at frequencies near the local value of 1/4 *f*_ce_. As constant-frequency wave elements propagate away from equator, frequencies near 1/4 *f*_ce_ catch up with lower frequency wave elements emitted at an earlier time in the initial frequency dispersed (chirped) emission generated at the equator. Second, the observed decrease in wave normal angle as the wave amplitude increases leads to a further increase in parallel propagation velocity for frequencies near ¼ *f*_ce_. Both these effects contribute to a “piling up” of wave power (analogous to “de-dispersion”) and an effective increase of d*f*/d*t* near 1/4 *f*_ce_ at the off-equator observing position.

For the strong subpackets we observe in these conditions, smooth wave frequency and phase variation with decreased but non-zero wave amplitude occur between subpackets (cf. Fig. 2). In Figs. 2 and 4, small frequency decreases are observed between these initial subpackets. This effect has been predicted in a recent paper by Hanzelka et al. (2020), who used nonlinear growth theory of chorus emissions to develop a simple model of subpacket formation.

**Fig. 7:**
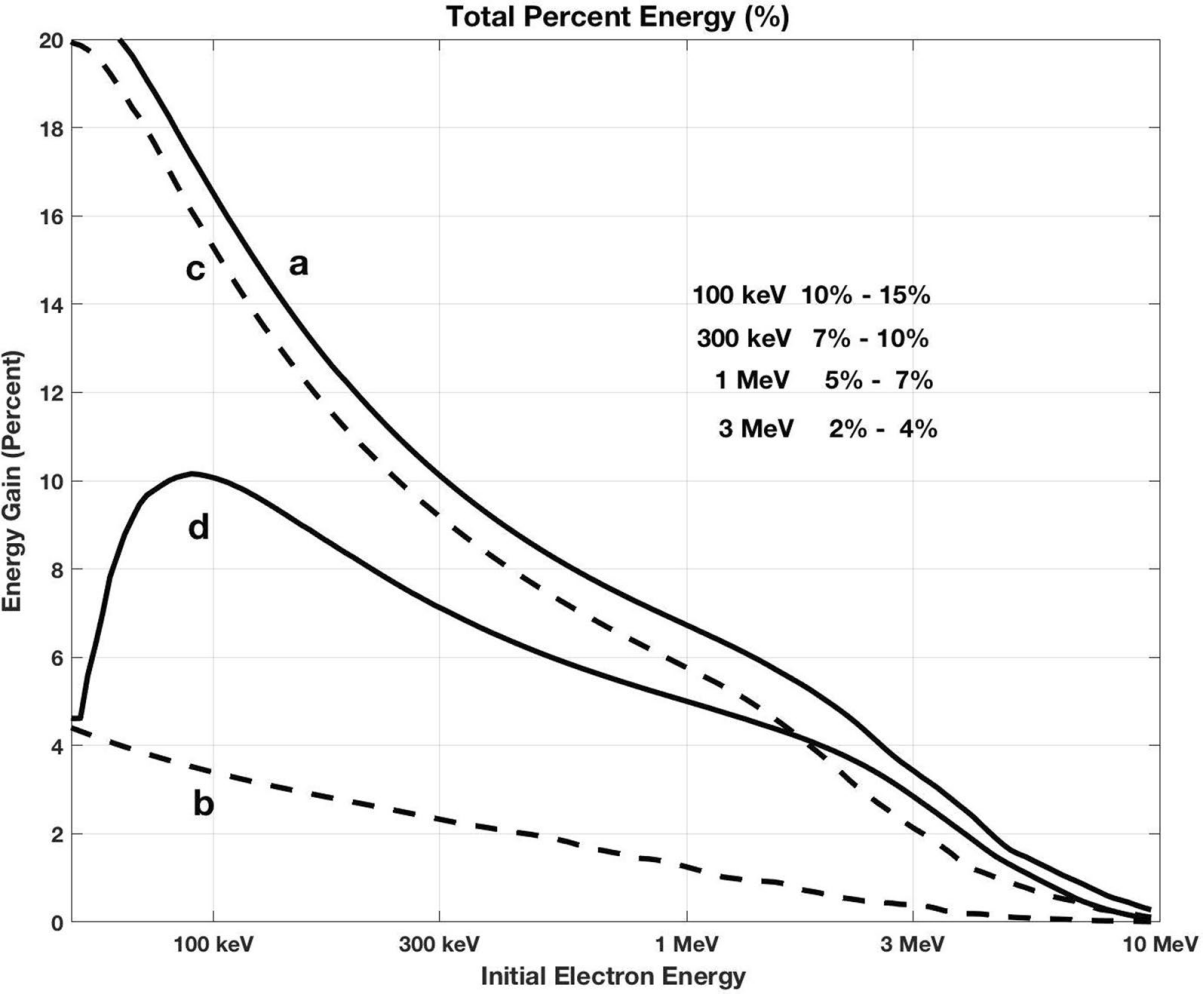
Percent energy gain is shown for seed electrons with initial energies between 30 keV and 10 MeV trapped in a single nonlinear interaction with the first two subpackets of the chorus elements shown in Fig. 2 Panels **a**–**d** following the calculations of Omura et al. (2019)

We note that there is little indication of discontinuous frequency jumps at the boundaries between these initial two subpackets observed during this event. This suggests good continuity of electron phase trapping between those subpackets along with increased overall potential for MeV acceleration (e.g., Higara and Omura 2020). Figure 7 plots the total percent energy gain for seed electrons trapped across both 1st and 2nd subpackets for the four chorus elements shown in Fig. 2. For the three chorus risers (a, c, and d) observed near *L* ~ 5 during the 16 UT radiation belt acceleration event, the total energy gain for individual seed electrons between 100 keV and 3 MeV ranges between 2 and 15%, obtained through their nonlinear interaction with a single chorus element.

There is a need for additional studies of the occurrence frequency of the type of strong subpacket structure we have described, and its relationship to the rapid recovery of the radiation belt electron population as occurred in the event we have presented here. Tsurutani et al. (2020) described rising-tone chorus subelements composed of coherent approximately “monochromatic” steps, with larger frequency variation at the beginning and ends of longer duration subelements. The frequency sweep rate discussed in Zhang et al. 2020 indicates the linear trend of the frequency variation of the chorus element. In that sense, the monochromatic subelements in Tsurutani et al. (2020) also have a finite frequency sweep rate corresponding to the average frequency variation approximated by a linear function. We present here cases where optimum wave growth takes place with rising-tone frequencies. The frequency variation is due to formation of resonant currents through nonlinear wave–particle interaction. The nonlinear dispersion relation of whistler-mode waves contains a term J_B_/B_w_, where J_B_ is a resonant current parallel to the wave magnetic field and B_w_ is the wave amplitude. When the growth of the subpacket saturates at a large amplitude, the *J*_B_/*B*_w_ becomes small, and the subpacket may look like a monotonic wave as described in Tsurutani et al. (2020). Both the smooth frequency increase and the gradient of the magnetic field are needed for the acceleration of energetic electrons. Since the higher energy particles with high pitch angles are mostly found near the equator where the gradient of the magnetic field is small, the frequency variation is especially important for acceleration of MeV electrons. For a monochromatic wave, acceleration is due to the gradient of the magnetic field. The acceleration of electrons trapped by the Landau resonance works effectively downstream from the equator in the presence of the magnetic field gradient because of the long interaction time for Landau resonant electrons moving in the same direction as the wave propagation (Hsieh et al. 2020).

**Fig. 8:**
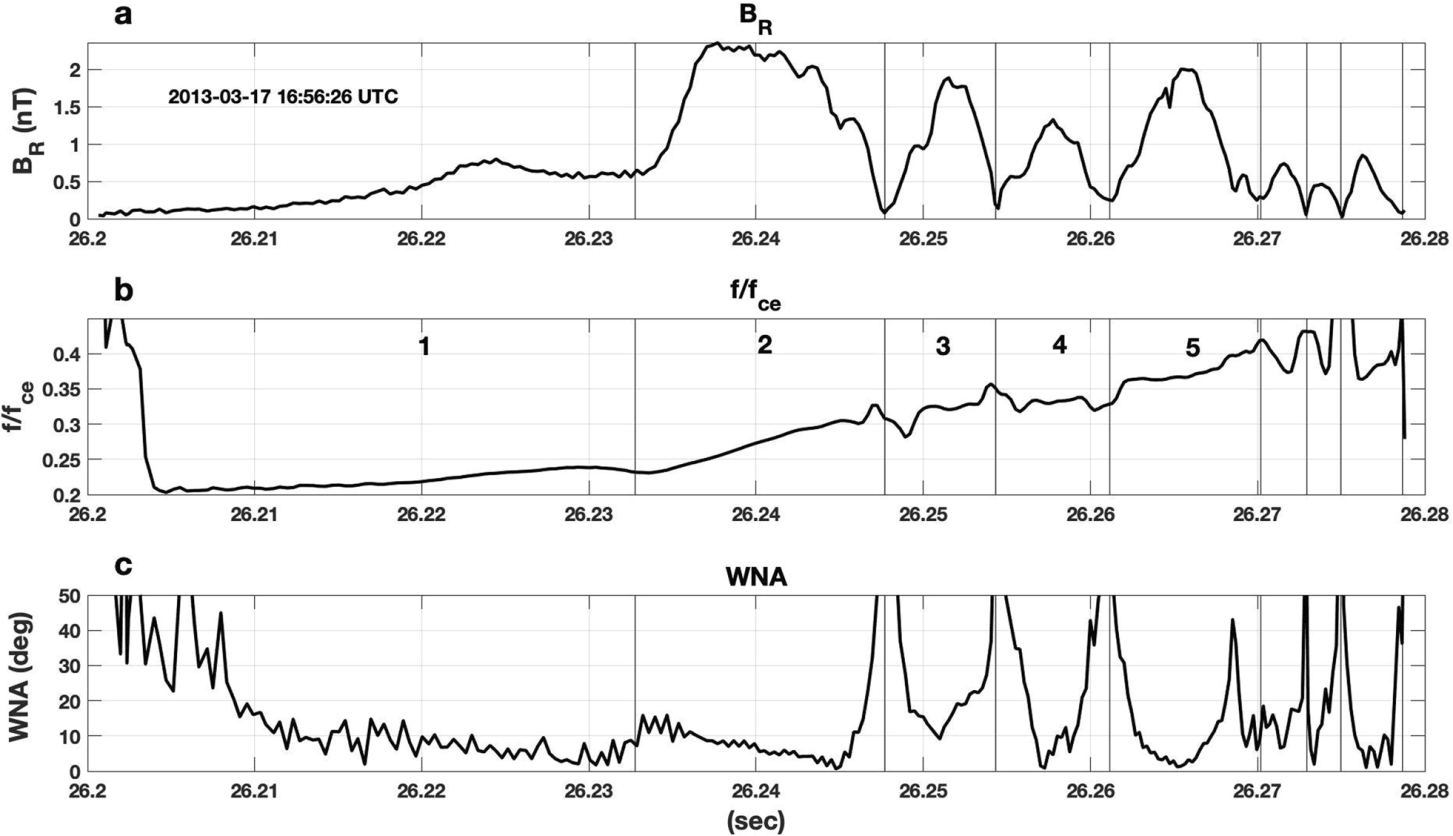
For the chorus element shown in Fig. 2a, **a** presents the right-hand mode wave magnetic field amplitude (*B*_r_), **b** normalized wave frequency, and **c** wave normal angle. The initial 5 subpackets are indicated with vertical lines. The long-duration subpackets (1, 2, and 5) exhibit smoothly rising frequency across their temporal extent while a step-like frequency plateau characterizes the shorter subpackets (3 and 4)

The subpackets shown here in Figs. 1, 2, and 4 exhibit a very clear frequency increase during the growth of individual subpackets. This is clearly seen for the strong 2nd subpacket in Fig. 2a where the wave amplitude saturates (|*B*_perp_|~ 2 nT) for ~ 8 ms while the wave frequency rises strongly and WNA decreases to < 5°. A possible explanation for this difference lies in the longer duration (> 10 ms) of the initial subpackets we study here. The statistical study of Zhang et al. (2020) noted that only 15% of the total wave power in strong chorus elements is carried in such long subpackets. In Fig. 8, we further examine the overall chorus element in Fig. 2a by showing an extended (80 ms) time interval including the first five subpackets. Right-hand whistler-mode wave magnetic field amplitude (*B*_r_), normalized wave frequency, and wave normal angle are shown. The initial 5 subpackets each reach maximum amplitude of 1 nT or greater. For this chorus element, the longer-duration subpackets (1, 2, and 5) exhibit smoothly rising frequency across their temporal extent, while a step-like frequency plateau characterizes the shorter subpackets (3 and 4). As noted earlier, when the chorus wave amplitude is small, stronger overlapping signals from other emissions could disrupt the wave cycle frequency leading to apparent large jumps in WNA (panel 8c) and frequency deviation (panel 8b) at such points.

## Summary and conclusions

Repeatable initial subpacket structure in strong chorus elements observed during rapid radiation belt acceleration events features coherent temporal durations of 10 to 30 ms and peak wave magnetic field amplitudes of 1–2 nT (*E*_R_ ~ 50–100 mV/m). As constant-frequency wave elements propagate away from the equator into regions of increasing magnetic field, damping at 1/2 *f*_ce_ progressively erodes wave amplitude at frequencies above 1/2 *f*_ceEQ_. Observed off the equator, an initial long (> 20 ms) coherent subpacket exhibits slowly rising frequency, wave normal angle < 20°, and frequency onset near 1/4 *f*_ceEQ_. A second stronger subpacket centered near 1/4 *f*_ce_ at the point of observation exhibits rapidly rising frequency (d*f*/d*t*) and small (< 10°) wave normal angle. Wave frequency and phase vary smoothly both within these 1st and 2nd subpackets and across the transition between them providing appropriate conditions for continuous resonant electron phase trapping, leading to good potential for MeV electron acceleration. Following the nonlinear equations of Omura et al. (2019), we calculate the energy gain of individual seed electrons trapped cycle-by-cycle with a full chorus wave subpacket. We note that the acceleration of electrons > 100 keV and the wave generation by electrons < 100 keV can be treated independently. Maximum energy gain of 5–10 keV/wave cycle is calculated for individual seed electrons with 1–3 MeV initial energy.

## Data Availability

Van Allen Probes observations used in this study can be obtained through instrument websites (EMFISIS wave data: http://emfisis.physics.uiowa.edu; MagEIS and HOPE particle data: https://rbsp-ect.lanl.gov/rbsp_ect.php. The values of electron energy gain shown in Figs. 6 and 7 are calculated from Eqs. (72) and (73) of Omura et al. (2019).

## Abbreviations

EMFISIS: Electric and Magnetic Field Instrument and Integrated Science
HOPE: Helium, Oxygen, Proton, and Electron mass spectrometer
MagEIS: Magnetic electron ion spectrometer
VLF: Very low frequency
WNA: Wave normal angle
